# Identification of telomere-related lncRNAs and immunological analysis in ovarian cancer

**DOI:** 10.3389/fimmu.2024.1452946

**Published:** 2024-09-17

**Authors:** Weina Xu, Shuliu Sang, Jun Wang, Shanshan Guo, Xiao Zhang, Hailun Zhou, Yijia Chen

**Affiliations:** ^1^ Department of TCM, Zhoujiadu Community Health Service of Shanghai Pudong New Area, Shanghai, China; ^2^ Department of Oncology, Yueyang Hospital of Integrated Traditional Chinese and Western Medicine, Shanghai University of Traditional Chinese Medicine, Shanghai, China; ^3^ Department of Gynecology, Longhua Hospital affiliated to Shanghai University of Traditional Chinese Medicine, Shanghai, China

**Keywords:** ovarian cancer, prognosis, telomere, immune, lncRNA

## Abstract

**Background:**

Ovarian cancer (OC) is a global malignancy characterized by metastatic invasiveness and recurrence. Long non-coding RNAs (lncRNAs) and Telomeres are closely connected with several cancers, but their potential as practical prognostic markers in OC is less well-defined.

**Methods:**

Relevant mRNA and clinical data for OC were sourced from The Cancer Genome Atlas (TCGA) database. The telomere-related lncRNAs (TRLs) prognostic model was established by univariate/LASSO/multivariate regression analyses. The effectiveness of the TRLs model was evaluated and measured via the nomogram. Additionally, immune infiltration, tumor mutational load (TMB), and drug sensitivity were evaluated. We validated the expression levels of prognostic genes. Subsequently, PTPRD-AS1 knockdown was utilized to perform the CCK8 assay, colony formation assay, transwell assay, and wound healing assay of CAOV3 cells.

**Results:**

A six-TRLs prognostic model (PTPRD-AS1, SPAG5-AS1, CHRM3-AS2, AC074286.1, FAM27E3, and AC018647.3) was established, which can effectively predict patient survival rates and was successfully validated using external datasets. According to the nomogram, the model could effectively predict prognosis. Furthermore, we detected the levels of regulatory T cells and M_2_ macrophages were comparatively higher in the high-risk TRLs group, but the levels of activated CD8 T cells and monocytes were the opposite. Finally, the low-risk group was more sensitive to anti-cancer drugs. The mRNA levels of PTPRD-AS1, SPAG5-AS1, FAM27E3, and AC018647.3 were significantly over-expressed in OC cell lines (SKOV3, A2780, CAOV3) in comparison to normal IOSE-80 cells. AC074286.1 were over-expressed in A2780 and CAOV3 cells and CHRM3-AS2 only in A2780 cells. PTPRD-AS1 knockdown decreased the proliferation, cloning, and migration of CAOV3 cells.

**Conclusion:**

Our study identified potential biomarkers for the six-TRLs model related to the prognosis of OC.

## Introduction

Ovarian cancer (OC) is the fifth most prevalent cancer globally (1, 2). Epithelial OC, accounting for the majority of cases, includes serous, endometrioid, mucinous, and clear cell subtypes, each with varying degrees of differentiation (3). The International Federation of Gynecology and Obstetrics (FIGO) classifies OC into five stages, ranging from stage 0 to extensive stage IV metastasis (4). Currently, targeted therapies, such as poly (ADP-ribose) polymerase inhibitors, and other treatments like surgery, radiation, immunotherapy, nanomedicine technology, and combinatorial therapies, have made rapid progress in improving outcomes for OC patients (5, 6). However, most OC patients still develop resistance, recurrence, metastasis, and the potential development of multi-system complications (7, 8). Hence, it is urgent to pinpoint novel biomarkers that can serve as predictors for clinical outcomes in OC.

Telomeres are special complexes crucial for protecting the ends of eukaryotic chromosomes (9). Abnormal telomeres can result in cancer and age-related pathologies which are related to the integrity of the DNA damage response (10). Notably, short telomeres contribute to genomic instability, which promotes cancer progression. Nevertheless, long telomeres may enhance the risk of cancer (11). It reported that telomerase is highly expressed in ovaries (12). Additionally, a multicenter study shows that the length of telomeres serves as the biomarker in elderly patients with OC (13).

Long non-coding RNAs (lncRNAs) with a length surpassing 200 nucleotides contribute to the modulation of chromatin dynamics, cellular growth, gene expression, differentiation, and developmental processes (14). Compared to mRNAs, lncRNAs are often less abundant in cells, exhibit more specialized tissue-specific expression, and can have a shorter lifespan (15). To fulfill their diverse biological roles, lncRNAs engage in intricate gene regulatory networks through interactions with mRNAs (16). Research has revealed that analyzing lncRNA-mRNA co-expression networks is instrumental for elucidating the involvement of lncRNAs in platinum resistance and for uncovering those with prognostic significance and therapeutic potential in OC (17, 18). Moreover, lncRNAs can modulate immune responses and serve as crucial prognostic biomarkers and diagnostic markers in OC (19, 20). Previous research revealed that certain lncRNAs, including TERC, TERRA, and GUARDIN, have been implicated in the intricate mechanisms of DNA damage response integration, safeguarding of telomere termini, and regulation of telomere length (21, 22). Indeed, emerging evidence suggests that telomere-related lncRNAs (TRLs) are associated with the prognosis of kidney renal clear cell carcinoma prognosis (23). However, the molecular underpinnings of how lncRNAs contribute to telomere homeostasis remain largely uncharted territory. We found that prior research has concentrated on the length and significance of telomeres in cancer prognosis, leaving the correlation of TRLs with OC unexplored. Recognizing the importance of both telomeres and lncRNAs, combining the two to improve prognosis in OC patients may be a viable strategy.

In the present study, we formulated TRLs from The Cancer Genome Atlas (TCGA) dataset to predict OC outcomes. Additionally, we probed the connection between the TRLs model and the immune and delved into the potential impact of TRLs on therapeutic drug selection. This study introduces novel perspectives and targets for treating OC.

## Methods

### Data collection

The transcriptome data, clinical information, and tumor mutational burden (TMB) information of 429 OC patients were from the TCGA (https://portal.gdc.cancer.gov/) database. Normal sample data (88 samples) were from the GTEx (http://commonfund.nih.gov/GTEx/) database. The annotation information was from the Ensembl database (https://www.ensembl.org) (24) to identify lncRNAs. The inclusion criteria for our study were strictly defined: (1) patients diagnosed with primary ovarian serous cystadenocarcinoma, (2) Samples with complete survival time and outcomes. After matching, a total of 370 OC samples were enrolled in the subsequent study and randomly divided into two groups (7:3 ratio), with 259 cases used for training and 111 cases for testing. The clinical baseline information of the patients is presented in **Supplementary Table 1**.

### Determination of TRLs

The 2093 telomere-related genes (TRGs) were downloaded from TelNet (http://www.cancertelsys.org/telnet/) database (25). The differentially expressed TRGs were pinpointed using the criteria of |log_2_ FC| ≥ 3 and FDR < 0.05 (23) with the “limma” package. We performed the Pearson correlation analysis to calculate the correlation coefficient between differentially expressed TRGs and lncRNAs expression using the “linkET” package. LncRNAs meeting *P* < 0.001 and |R|> 0.7 (18) screening criteria were designated as TRLs.

### Establishment and validation of the prognostic model

To identify potential prognostic TRLs in OC patients, the univariate Cox analysis was utilized to integrate the TRLs expression information with survival information using the “survival” package. The least absolute shrinkage and selection operator (LASSO) analysis could mitigate the impact of multicollinearity among the numerous genetic variables (26). This approach, when synergized with the Cox model, can refine the screening of potential biomarkers (27). In the training cohort, we conducted a LASSO analysis to filtrate the prognostic TRLs ulteriorly using the “glmnet” package. The LASSO-selected data applied the multivariate Cox analysis to identify which TRLs emerged as independent prognostic factors for OC patients (28–31). Subsequently, Kaplan-Meier (KM) analysis was utilized to contrast the high- and low-risk groups, divided by the median risk score. The KM method generated a survival curve with time depicted on the x-axis and the cumulative survival probability represented on the y-axis, offering a visual representation of the proportion of individuals surviving over the study period. If the log-rank test p-value is less than 0.05, there is a statistically significant difference between survival curves (32). Additionally, Receiver Operating Characteristic (ROC) analysis was applied to observe the predictive value of the TRLs prognostic model using the “timeROC” package. The ROC curve was plotted with the false positive rate on the x-axis and the true positive rate on the y-axis, featuring a 95% confidence interval indicated by dotted lines. The area under the ROC curve (AUC) quantified the model’s discriminatory ability, the AUC value closer to 1.0 signified superior model performance and generalization capabilities (33, 34). To assess the independence of risk scores derived from the signature, we performed the Cox regression analyses. Subsequently, we verified the prediction performance using testing and entire sets. Ultimately, the integrated nomogram was constructed, encompassing all independent prognostic parameters using the “rms” package, to provide a qualitative prediction of the overall survival (OS) for OC patients within the entire set.

### Go and KEGG analysis

To explore the functional mechanisms associated with the model, we determined the differentially expressed genes between low- and high-risk TRLs groups (|log_2_FC| > 1 and FDR < 0.05) using the “limma” package. Subsequently, we conducted GO and KEGG analyses on the above genes. *P* < 0.05 was considered significant.

### Immune profile analysis

To delve into the connection between risk score and immune infiltration, the ESTIMATE algorithm was employed to compute the abundance of stromal and immune components. Patients were stratified into two groups according to the median values of risk score levels. Then, the abundance of 28 immune-cell types in two groups was determined by the ssGSEA. Utilizing the CIBERSORT algorithm, we estimated the abundance of 22 distinct immune cell subtypes in patients. The distribution pattern of the two groups was visualized by PCA analysis. To investigate the correlation between immune cells and prognostic TRLs, the results were performed by the Pearson correlation analysis and visualized utilizing the “ggplot2” package.

### Tumor mutation analysis

The TMB data was computed in low- and high-TMB groups, categorized by the median TMB value of OC patients. Subsequently, the mutational landscape was depicted in a waterfall plot using the “maftool” package. Furthermore, comparative and survival analyses were executed to explore disparities in somatic mutations between the two groups.

### Prediction of potential drug sensitivity

The “oncoPredict” package was employed to forecast drugs IC_50_ values. The IC_50_, indicative of tolerance capacity, was computed, with higher IC_50_ values signifying increased resistance of cells to the drugs. Subsequently, the disparity in sensitivity scores between the two groups was analyzed.

### Cell culture

OC cell lines (SKOV3, A2780, CAOV3) and IOSE-80 cells were sourced from the Cell Bank of the Chinese Academy of Sciences. SKOV3 cells were grown in McCoy’s 5A medium (Servicebio Technology Co., Ltd., China, G4541-500ML) with 10% fetal bovine serum. A2780 cells and IOSE-80 cells were grown in RPMI-1640 medium (Servicebio Technology Co., Ltd., China, G4535-500ML) with 10% fetal bovine serum. CAOV3 cells were grown in DMEM medium (Servicebio Technology Co., Ltd., China, G4515-500ML) with 10% fetal bovine serum. Then, the cells were cultivated in a 37°C incubator with 5% CO_2_.

### qRT-PCR validation

Total RNA was extracted from the cells using an RNA extraction solution (Servicebio Technology Co., Ltd., China, G3013-100ML). For the conversion of RNA to cDNA, we utilized a cDNA Synthesis kit ((TransGen, China, AU341-02) to carry out the reverse transcription process. The quantification of lncRNA levels was determined using the 2^-ΔΔCq^ method, with the results being normalized against the expression levels of the GAPDH. The TRLs sequences are shown in **Table 1**.

**Table 1:** The TRLs sequences.

Gene Name	Forward sequence (5’-3’)	Reverse sequence (5’-3’)
PTPRD-AS1	CTATTCATCATCACCTCCACATTC	AATTATGCACTAGAGGGGGTAGTG
SPAG5-AS1	CTCTCAGATCACCACATTGTTTTC	TAAGTCTGATGACACAGCAGAACA
CHRM3-AS2	GAGTCTAGCATCTTGCATCTTCCT	TGTTGAGGATAGAACTAGCACAGC
AC074286.1	CCACTGCCAGTTAGAAGACCTATT	AGATCAGCACCACATACACCTAAA
FAM27E3	CACTTGAGAAACAGACCGTATTGT	CTAGGATCAAGATGAACACACTGC
AC018647.3	AGTATACACTGCACCCTGTTTGTG	ACCTGGATGAGACTGGAGACTATT
GAPDH	TGACAACTTTGGTATCGTGGAAGG	AGGCAGGGATGATGTTCTGGAGAG

### Transfection

CAOV3 cells, in the exponential growth phase, were seeded in a six-well plate at a density of 4 × 10^4^ cells per well to achieve 70–80% confluency. Subsequently, CAOV3 cells were transfected with small interfering RNA targeting PTPRD-AS1 (si‐PTPRD-AS1) and a non-targeting siRNA as a negative control (siNC) using the Lipofectamine 2000 reagent (Thermo Fisher Scientific, USA, 11668-027). The PTPRD-AS1 siRNAs, siNC, and Lipofectamine 2000 were prepared by dilution in Opti-MEM serum-free medium (Gibco, USA, 31985-070), then combined and allowed to incubate for 20 minutes to form the transfection complex. This mixture was subsequently introduced into CAOV3 cells for transfection. After a 6-hour incubation period, the medium was replaced with the fresh complete growth medium. The CAOV3 cells were then collected 48 h post-transfection for further analysis. To confirm the knockdown efficiency, qRT-PCR analysis was conducted post-transfection. The sequences of siRNAs are shown in **Table 2**.

**Table 2:** The PTPRD-AS1 gene siRNA sequence.

Gene Name	Sense(5’-3’)	Antisense(5’-3’)
PTPRD-AS1 si-NC	UUCUCCGAAGGUGUCACGUTT	UUCUCCGAAGGUGUCACGUTT
PTPRD-AS1 si-1	GCUCUAACAUUCUGUGAUATT	UAUCACAGAAUGUUAGAGCTT
PTPRD-AS1 si-2	CGAUGACAAUAAUAGUAAUTT	AUUACUAUUAUUGUCAUCGTT

### CCK8 and colony formation assay

The CCK8 assay and the colony formation assay were utilized to determine the effect of PTPRD-AS1 knockdown on the proliferation of CAOV3 cells. 4,000 CAOV3 cells transfected with si-NC or si-PTPRD-AS1 per well were seeded into 96-well plates and grown for 48 h. Subsequently, the optical density (OD) values were measured at 450 nm within 1-4 h after the addition of 10 μL/well of the CCK-8 reagent (Beyotime Inc., China, C0038). For colony formation, a total of 1,000 CAOV3 cells transfected with si-NC or si-PTPRD-AS1 per well were dispensed into 6-well plates and cultured for 2 weeks. Upon the emergence of visible colonies, the cells were processed for staining using crystal violet (Beyotime Inc., China, C0121).

### Transwell assay

Upper transwell chambers coated with matrigel (Corning, USA, 354234) were utilized to assess the invasive capacity of PTPRD-AS1 knockdown. 2×10^4^ CAOV3 cells transfected with si-NC or si-PTPRD-AS1 were plated in the upper chambers with DMEM medium devoid of FBS. The lower chambers were filled with DMEM supplemented with 10% FBS. Subsequently, the cells were fixed using a 4% paraformaldehyde solution for 30 minutes and stained with crystal violet for an additional 30 minutes to facilitate the quantification of invasive cells.

### Wound healing assay

The assessment of cell migration was conducted using a wound healing assay. CAOV3 cells transfected with si-NC or si-PTPRD-AS1 were plated in 6-well plates to achieve 80% confluence. A standardized scratch wound was created across the well using a 10 μL pipette tip to generate a uniform gap and photographed after 24 h.

### Statistical analysis

All statistical analyses were carried out through R software (version 4.3.1) and GraphPad Prism 8.0 software. Two Group differences were statistically evaluated using the Student’s t-test. For the analysis of multiple groups, One-way ANOVA was the procedure followed. *P* < 0.05 was considered significant.

## Results

### Determination of TRLs

**Figure 1:**
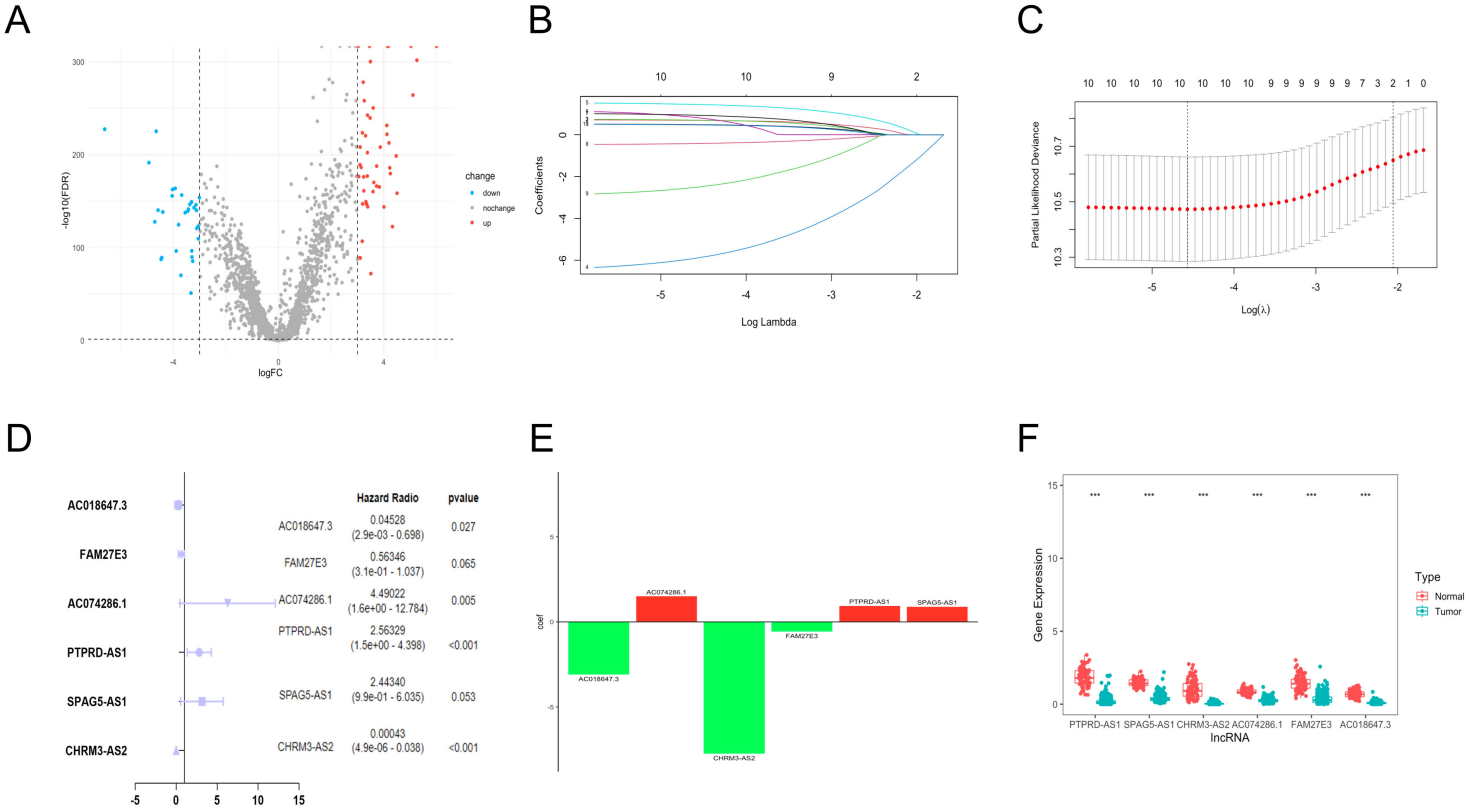
Establishment of TRLs prognostic model in OC. **(A)** The differentially expressed TRGs between normal and OC samples. **(B)** The distribution of lambda values in LASSO analysis. **(C)** The deviance diagram of LASSO analysis. **(D)** The six prognostic TRLs by multivariate analysis. **(E)** The coefficient distributions of six prognostic TRLs. **(F)** The expression of six prognostic TRLs between normal and OC tissues. *** *P* < 0.001.

We obtained the expression information of 2093 TRGs. 79 differentially expressed TRGs, including 31 down-regulated and 48 up-regulated genes (**Figure 1A**), and 1,6901 lncRNAs were determined. Finally, 77 TRGs were reserved, and 381 lncRNAs were determined as TRLs (|R| > 0.7 and *P* < 0.001).

### Establishment of TRLs prognostic model

A total of 10 TLRs of 381 TRLs were observably connected with OS via the univariate analysis (**Supplementary Table 2**). LASSO analysis was performed according to the 10 TRLs, and no genes were screened for incompatibility (**Figures 1B, C**). Subsequently, we performed the multivariate Cox regression analysis and identified six key TLRs (**Figure 1D**). The risk score for prognostic-related TRLs = (0.941293163 × PTPRD-AS1) + (0.893391681 × SPAG5-AS1) + (-7.750446273 × CHRM3-AS2) + (1.50190086 × AC074286.1) + (-0.57365439 × FAM27E3)+ (-3.094785104 × AC018647.3). Notably, CHRM3-AS2, FAM27E3, and AC018647.3 were protective factors with HR < 1 in OC patients. Nevertheless, PTPRD-AS1, SPAG5-AS1, and AC074286.1 were risk factors with HR > 1. The findings indicated that PTPRD-AS1, CHRM3-AS2, AC074286.1, and AC018647.3 were independent prognostic factors of OC patients (*P*< 0.05). The coefficient results of six TRLs are shown in **Figure 1E**. The expression levels of six TLRs in tumor cases in comparison to normal cases are shown in **Figure 1F**.

### Validation of the TRLs prognostic model

**Figure 2:**
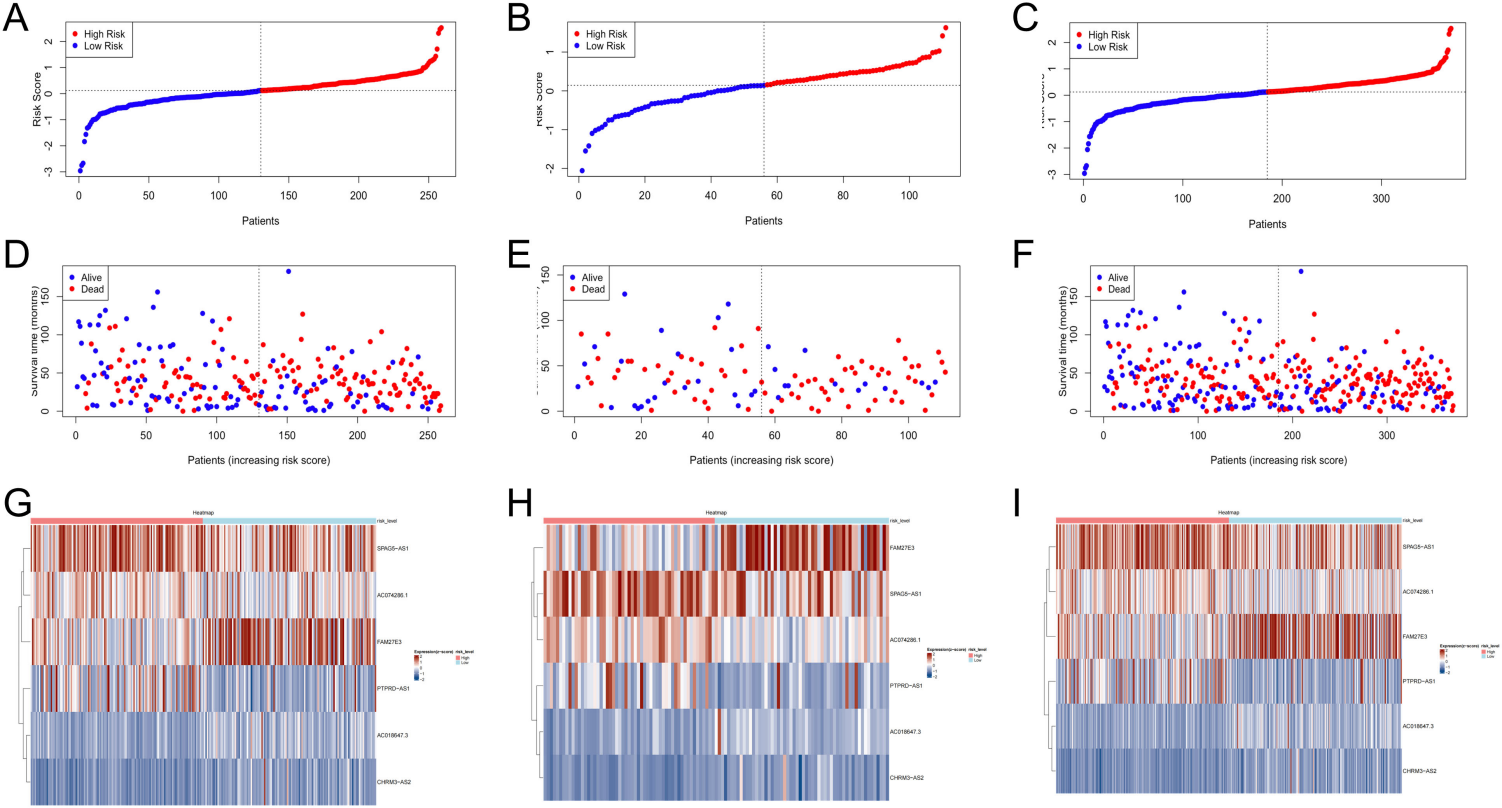
Verification of prognostic model in the training, testing, and overall sets. **(A–C)** The risk scores. **(D–F)** The survival status. **(G–I)** Heatmaps of 6 TRLs expression.

**Figure 3:**
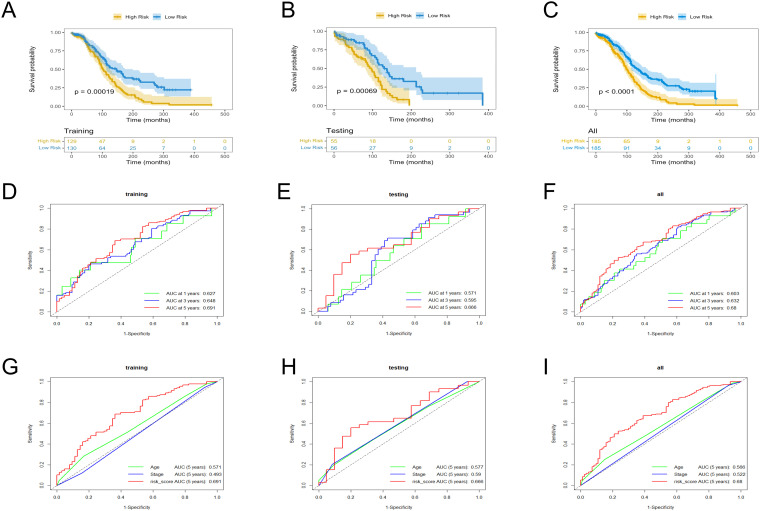
KM and ROC curves analysis in the training, testing, and overall sets. **(A–C)** OS rates. **(D–F)** ROC curves for the prognostic model. **(G–I)** ROC curves for the six TRLs risk scores and clinical factors.

The low- and high-risk groups were categorized by the median score in the training, testing, and entire cohorts (**Figures 2A–C**). Analysis of survival status distributions indicated a higher mortality rate in the high-risk group (**Figures 2D–F**). The six TRLs in two groups were visually depicted through heat maps (**Figures 2G–I**). In the high-risk group, PTPRD-AS1, SPAG5-AS1, and AC074286.1 demonstrated comparatively elevated expression levels, while three protective lncRNAs (PTPRD-AS1, SPAG5-AS1, and AC074286.1) displayed the opposite trend. Additionally, the low-risk group experienced a higher OS rate (**Figures 3A–C**). The precision of the TRLs prognostic model was assessed by ROC analysis, revealing that the TRLs prognostic signature demonstrated potential in predicting OS (training cohort: 5-year AUC = 0.691, test cohort: 5-year AUC = 0.666, and entire cohort: 5-year AUC = 0.68) (**Figures 3D–F**). Furthermore, the risk score demonstrated superior prognostic prediction for OC patients compared to other clinical variables (**Figures 3G–I**). PCA analysis was employed to distinct distribution patterns between the two risk groups in the training, testing, and overall sets (**Supplementary Figure 1**). The two risk groups tended to diverge along two paths. These results underscored the robust predictive capability of this signature.

### Identification of TRLs prognostic model independence

**Figure 4:**
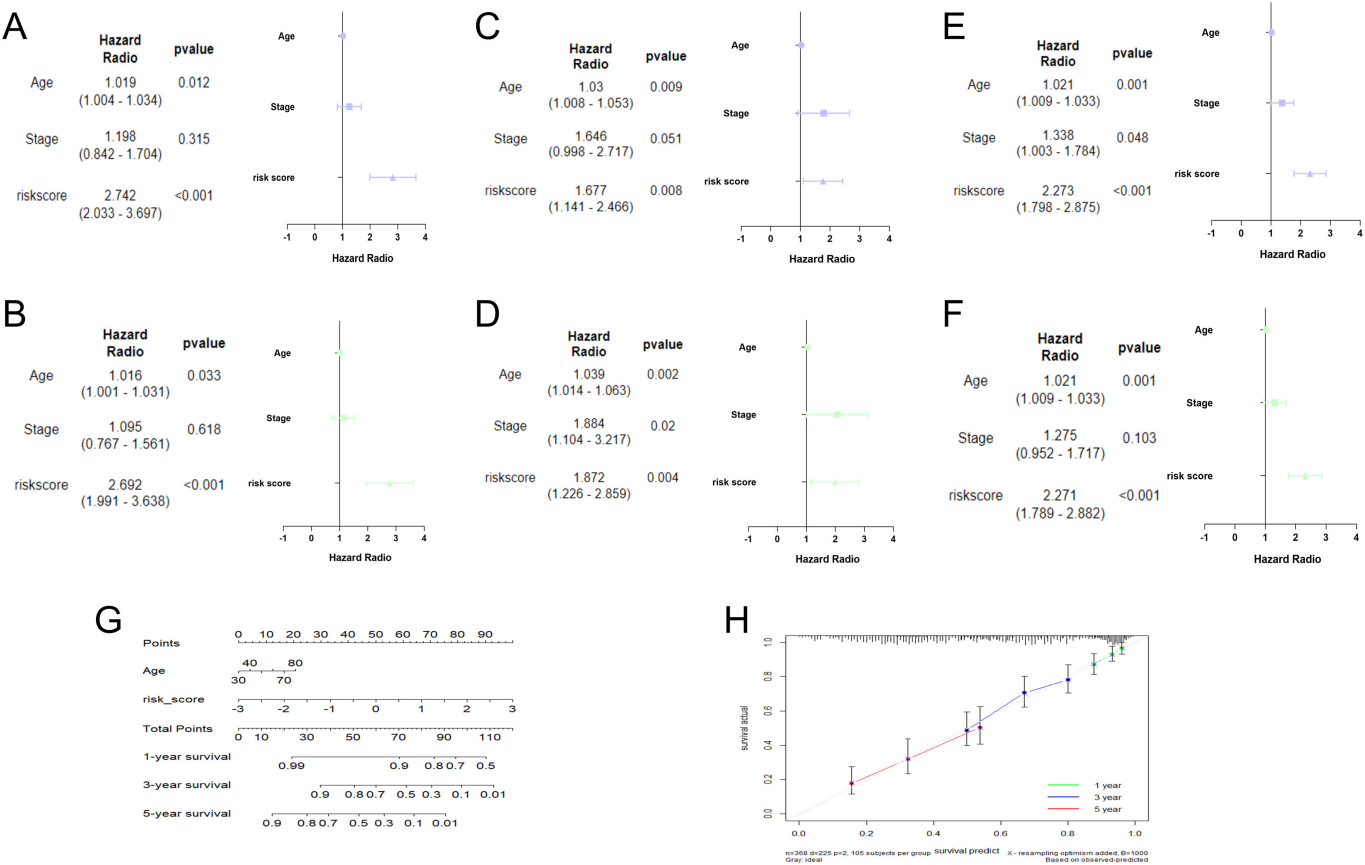
Establishment of nomogram. Univariate and multivariate analysis in the training **(A, B)**, testing **(C, D)**, and overall **(E, F)** sets. **(G)** Nomogram analysis in OC patients. **(H)** The calibration curves of the nomogram.

In the training cohort, univariate analysis showed risk score was related to patient survival (**Figure 4A**). Furthermore, the multivariate analysis suggested that risk score was the independent prognostic factor for OC patients (**Figure 4B**). The analysis results for both the testing set (**Figures 4C, D**) and the overall set (**Figures 4E, F**) were consistent. Subsequently, a nomogram was conducted by combining the risk score with clinical features (**Figure 4G**). The calibration curves proved the dependability of the six TRLs prognostic model (**Figure 4H**).

### Enrichment analysis

**Figure 5:**
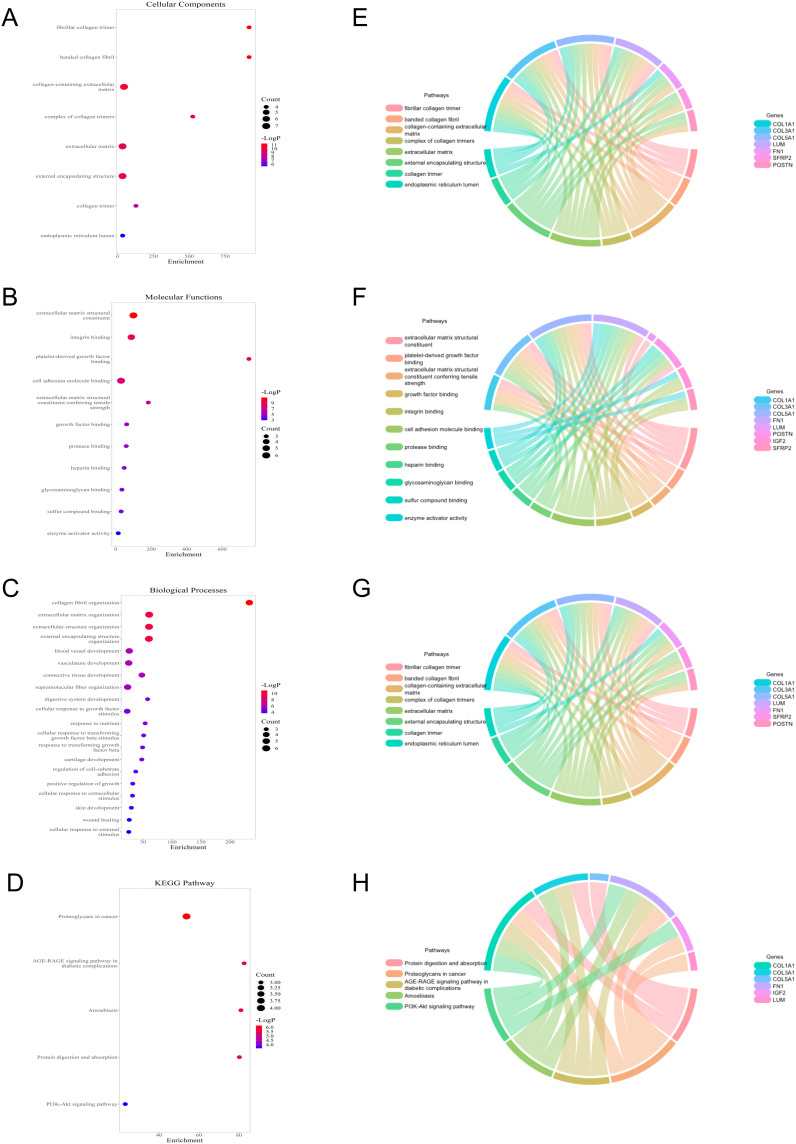
Functional analysis of 12 genes with differential expression between low- and high-risk groups. **(A–C)** GO terms analysis. **(D)** KEGG pathways analysis. **(E–H)** Specific genes related to the GO terms and pathways.

To study the potential biological function between the two groups, relevant enrichment analyses were carried out with 12 genes of differential expression between the two groups. GO terms showed that 12 genes were related to fibrillar collagen trimer and collagen fibrill organization (**Figures 5A–C**), while the KEGG pathway contained proteoglycans in cancer and PI3K-Akt signaling pathway, etc. (**Figure 5D**). Moreover, the cnetplots were used for the specific GO terms and KEGG categories (**Figures 5E–H**). These biological processes and pathways likely contribute to the high-risk group towards poorer clinical prognosis.

### Immune infiltration analysis

**Figure 6:**
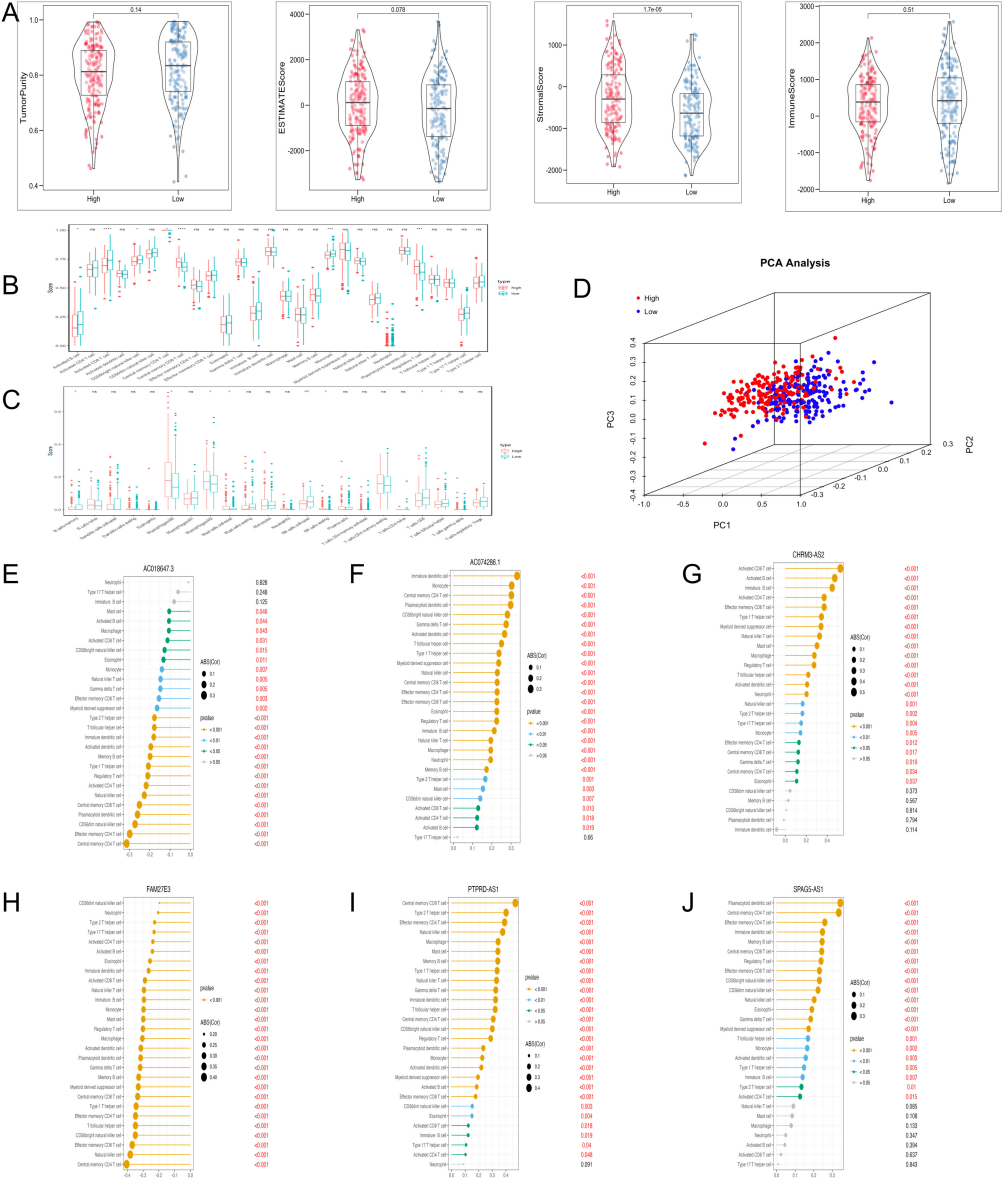
Immune cell infiltration analysis with TRLs risk group. **(A)** The ESTIMATE score, Immune score, and Stromal score diagrams. **(B)** The 28 type immune cells in two risk groups by ssGSEA. **(C)** The level of 22 immune cell infiltration in two risk groups by the CIBERSORT algorithm. **(D)** PCA analysis of immune cells between two groups. **(E–J)** Correlation analysis of six prognostic biomarkers (PTPRD-AS1, SPAG5-AS1, CHRM3-AS2, AC074286.1, FAM27E3, and AC018647.3) and immune cells. * *P* < 0.05, ** *P* < 0.01, **** *P* < 0.0001, ns, not significant.

The levels of stromal score were significantly lower in the low-risk group (**Figure 6A**). ssGSEA analysis demonstrated that the abundance of regulatory T cells was comparatively higher in the high-risk group, and the abundance of activated CD8 T cells and monocytes was the opposite (**Figure 6B**). CIBERSORT analysis revealed that the high-risk group had significantly higher levels of M_2_ macrophages (**Figure 6C**). PCA plot demonstrated the marked classification of immune cells in two groups (**Figure 6D**). Moreover, the correlation analysis indicated that PTPRD-AS1, SPAG5-AS1, CHRM3-AS2, AC074286.1, FAM27E3 and AC018647.3 were strongly associated with 28 immune cells (**Figures 6E–J**). In summary, these findings reveal the obvious differences between the two groups in the immune cells and are closely related to PTPRD-AS1, SPAG5-AS1, CHRM3-AS2, AC074286.1, FAM27E3, and AC018647.3.

### Tumor mutation analysis

**Figure 7:**
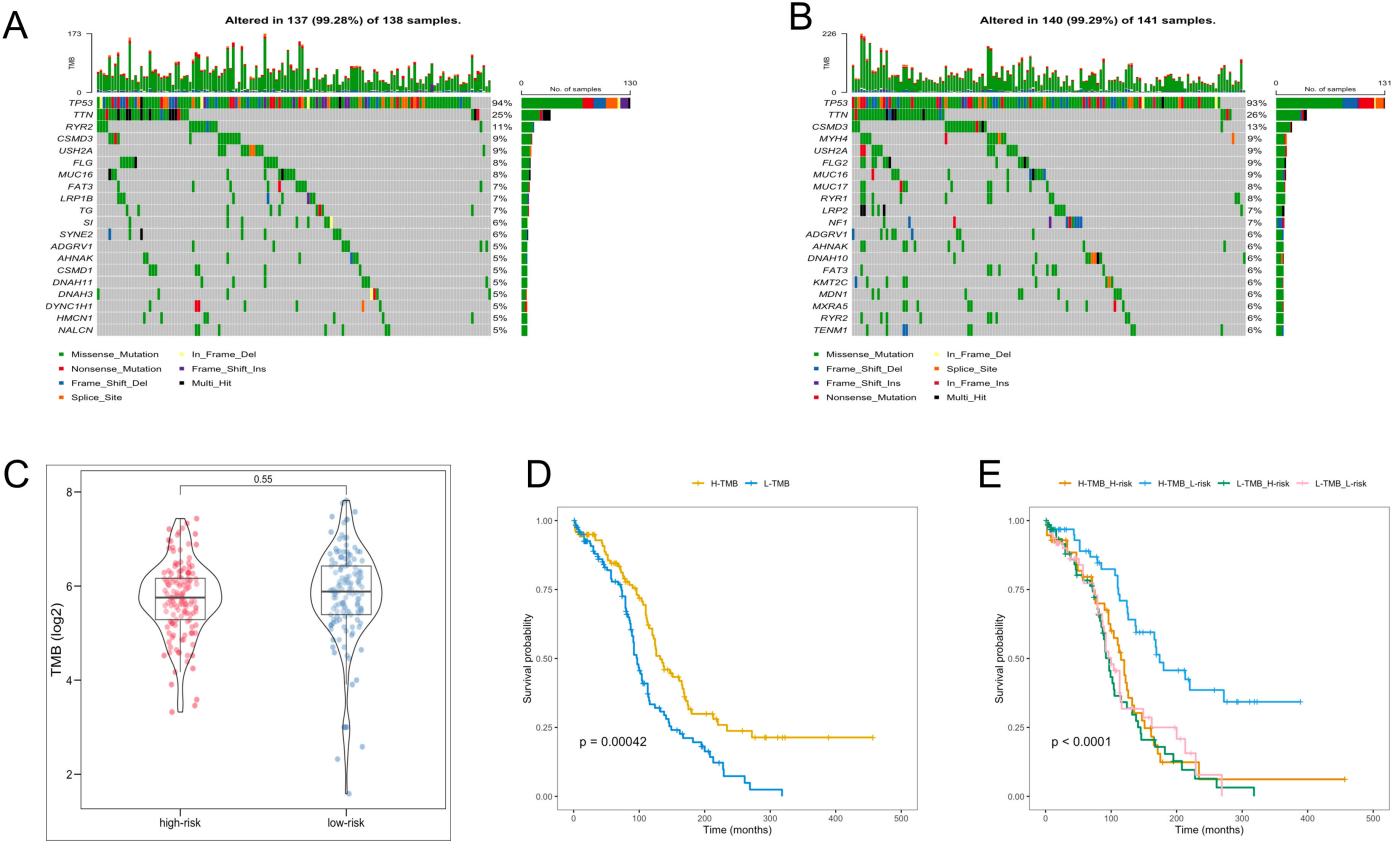
TMB analysis. **(A, B)** The waterfall plot of the frequency of TMB in two risk groups. **(C)** TMB levels between the two risk groups. **(D)** KM analysis of TCGA-OV patients in the low- and high-TMB groups. **(E)** KM analysis of TCGA-OV patients in the two risk groups combining the TMB groups.

In the analysis of TMB in OC patients, individual TMB values were determined. The probability of TMB in the high- and low-risk groups was 99.28% and 99.29%, respectively (**Figures 7A, B**). Nevertheless, the TMB values between the two groups were no significant difference (**Figure 7C**). Subsequent survival analyses were conducted across different TMB and risk groups. Notably, the high-TMB group exhibited the favorable OS, and the high-TMB combined with the low-risk group demonstrated the highest OS rate (**Figures 7D, E**).

### Sensitivity of potential drugs

**Figure 8:**
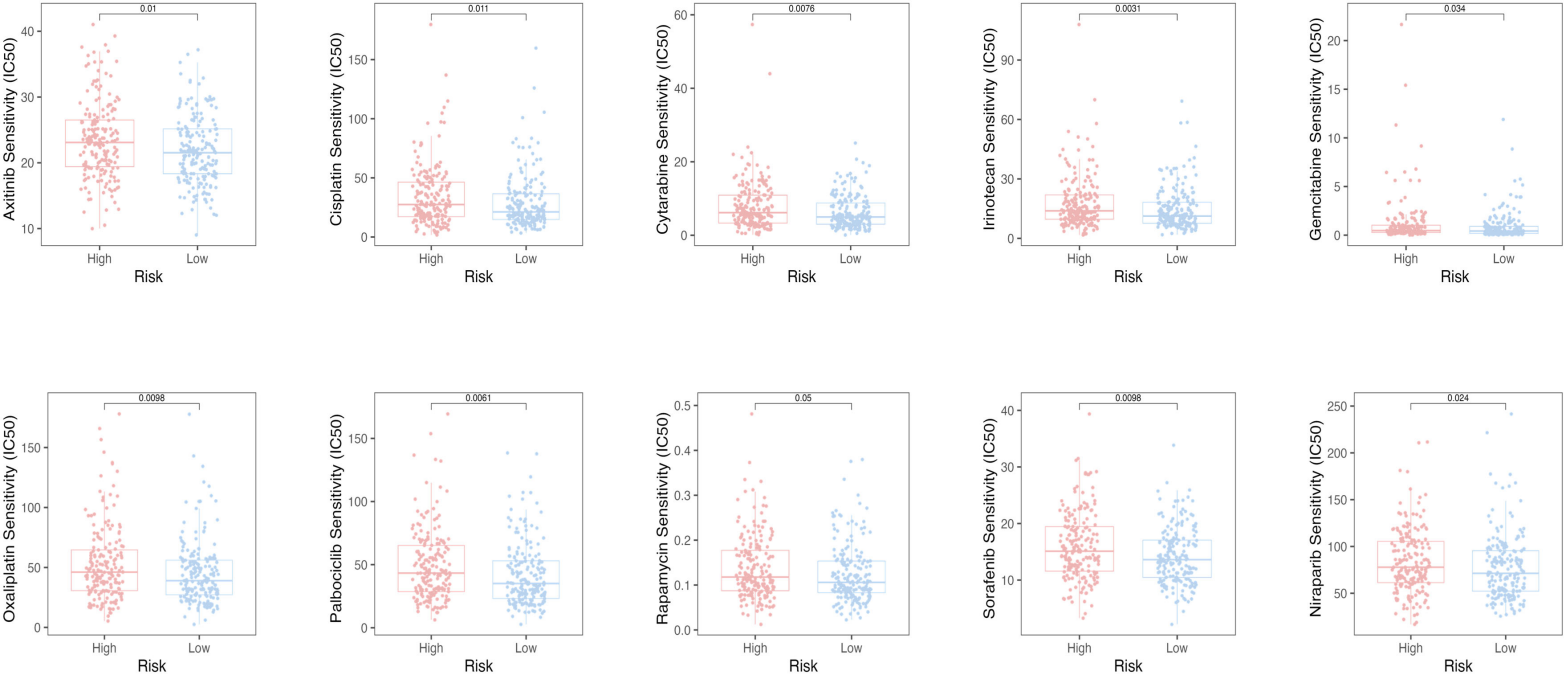
The oncoPredict algorithm predicted the IC_50_ values for ten anti-cancer drugs.

To assess the efficacy of the TRLs prognostic model for predicting several drugs in OC, we calculated the IC_50_ value of the anti-cancer drugs in two groups of OC patients. The low-risk group had the low IC_50_ value of the anti-cancer drugs, which means that the above drugs were more sensitive to the low-risk group (**Figure 8**).

### PTPRD-AS1 associated with OC prognosis

**Figure 9:**
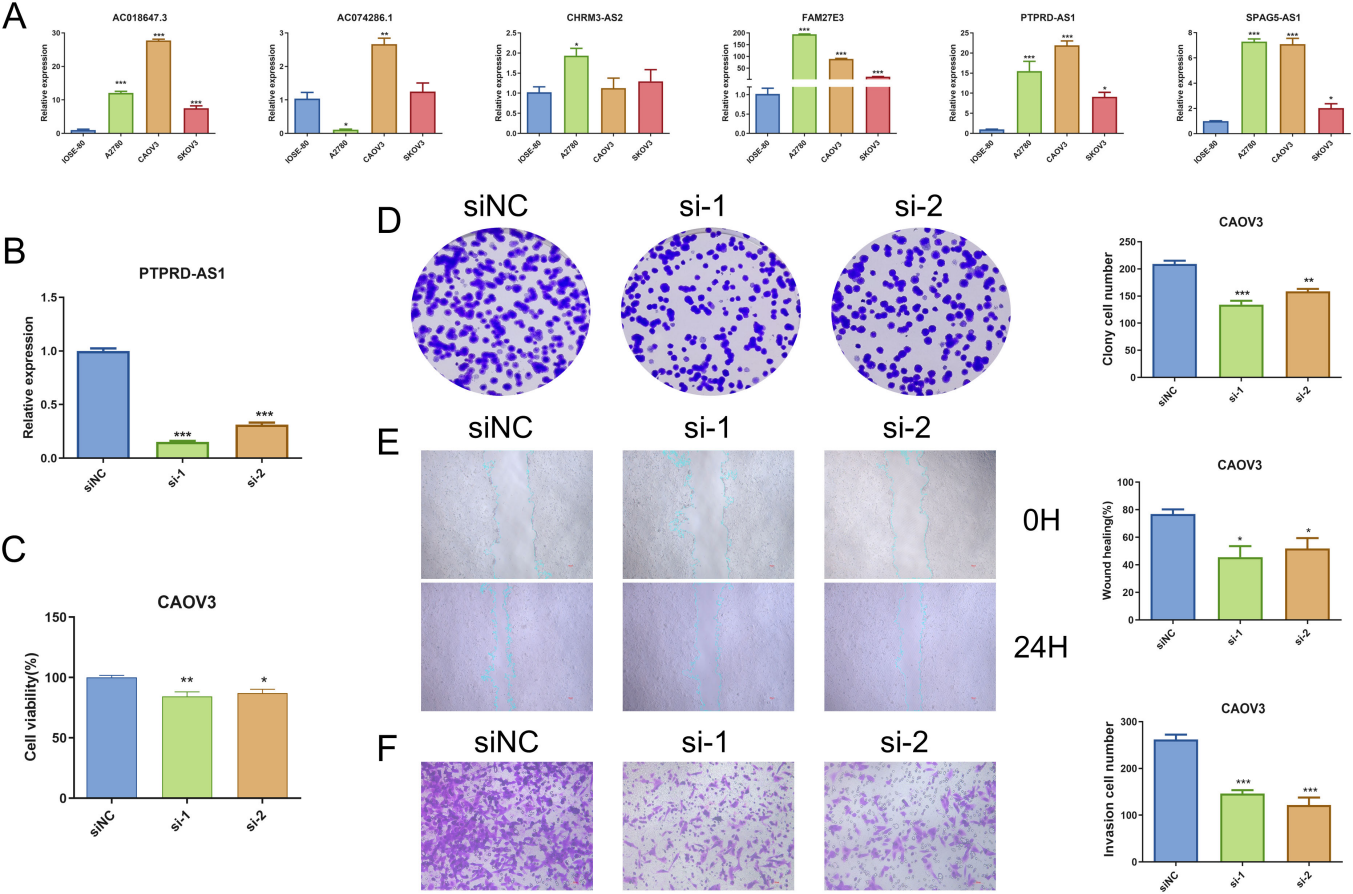
Analysis of PTPRD-AS1 mRNA Levels in OC. **(A)** Comparative expression levels of six prognostic genes in OC cell lines (SKOV3, A2780, CAOV3) and normal IOSE-80 cells by qRT-PCR. **(B)** Expression levels of PTPRD-AS1 in CAOV3 cells transduced with siRNA by qRT-PCR. **(C–F)** CCK8 assay, colony formation assay, wound healing assay, and transwell assay in transduced CAOV3 cells. The experiments were repeated at least three times. The One-way ANOVA was applied for statistical significance, * *P* < 0.05, ** *P* < 0.01, *** *P* < 0.001.

We performed qRT-PCR, and the result demonstrated that PTPRD-AS1, SPAG5-AS1, FAM27E3, and AC018647.3 were significantly over-expressed in OC cell lines (SKOV3, A2780, CAOV3) in comparison to normal IOSE-80 cells (*P* < 0.05). AC074286.1 were over-expressed in A2780 and CAOV3 cells and CHRM3-AS2 only in A2780 cells (*P* < 0.05) (**Figure 9A**). FAM27E3 had the highest expression in OC cell lines. We found that high expression of FAM27E3 was associated with a good prognosis (*P* = 0.036). Moreover, the levels of FAM27E3 were increased in stages III and grade III in OC. However, there was no significant difference among different stages and grades (**Supplementary Figure 2**). Among them, PTPRD-AS1, CHRM3-AS2, AC074286.1, and AC018647.3 were independent prognostic factors of OC patients. PTPRD-AS1 was strongly associated with the immune cells. In addition, high expression levels of PTPRD-AS1 have been proven to be associated with shorter survival of OC (35). But there is no further experimental evidence. Thus, we selected PTPRD-AS1 for more in-depth analysis. We utilized OS as the outcome measure to evaluate its effect on prognostic outcomes. Our results demonstrated a significant correlation between elevated PTPRD-AS1 expression and a poorer prognosis (*P* = 0.036, **Supplementary Figure 3A**), which was consistent with the previous research. Concurrently, we appraised the anti-cancer drugs sensitivity analysis. Patients exhibiting high levels of PTPRD-AS1 showed a more favorable response to the drugs Rapamycin and Dasatinib (**Supplementary Figures 3B–F**).

### PTPRD-AS1 inhibition suppresses CAOV3 cells proliferation, migration, and invasion

PTPRD-AS1 exhibited the highest mRNA expression levels in CAOV3 cells compared to other OC cell lines. Consequently, CAOV3 cells were chosen for further functional analysis. We achieved successful knockdown of PTPRD-AS1 in CAOV3 cells through transfection with Si-PTPRD-AS1 (**Figure 9B**). Importantly, the CCK8 assay and colony formation assay demonstrated a reduction in the proliferative capacity of CAOV3 cells after the knockdown of PTPRD-AS1, respectively (**Figures 9C, D**). Additionally, our findings indicated that the suppression of PTPRD-AS1 significantly impeded the metastatic and invasive properties of CAOV3 cells (**Figures 9E, F**). Notably, qPCR experiments revealed a significant downregulation of MMP2 and MMP9 upon PTPRD-AS1 knockdown (**Supplementary Figure 4**). Collectively, these results imply that the knockdown of PTPRD-AS1 exerts an inhibitory effect on the proliferation, migration, and invasion of CAOV3 cells.

## Discussion

OC remains a global malignancy characterized by metastatic invasiveness and recurrence (36). Notably, prognosis models based on lncRNAs have demonstrated accurate predictions for OC patient outcomes (37, 38). Although the clinical application of lncRNAs as predictive biomarkers is not yet fully understood, they offer distinct advantages over protein and mRNA biomarkers due to their tissue and stage-specific expression (39). This calls for more research to identify similar biomarkers for OC. Telomeres play a crucial role in preserving genomic integrity by safeguarding chromosome ends (40). Interestingly, anomalies in telomeric structures have been associated with various cancer types, underscoring their impact on oncogenesis and tumor progression. However, few studies explore the links between telomeres and lncRNAs in OC.

In this study, 79 TRGs were initially screened for differential expression between normal samples and OC samples. Through univariate Cox regression analysis, 10 TRLs were identified. Subsequently, based on LASSO and multivariate analysis, the prognostic model comprising 6 TRLs (PTPRD-AS1, SPAG5-AS1, CHRM3-AS2, AC074286.1, FAM27E3, and AC018647.3) was constructed. Among them, PTPRD-AS1 has been recognized as an effective biomarker for predicting the prognosis of breast cancer patients and OC patients (41). SPAG5-AS1 can promote apoptosis to improve and alleviate podocyte injury (42). CHRM3-AS2 was highly expressed in glioma cells, and silencing of CHRM3-AS2 expression could inhibit glioma growth (43). AC074286.1 had been proven as a protective lncRNA in glioma, and its high expression showed a favorable prognosis (44). The expression of FAM27E3 was up-regulated in thyroid cancer, and the high expressions of FAM27E3 suggested poor prognosis (45). However, the role of AC018647.3 remains unexplored in the literature, so future studies will necessitate investigations to unravel its functions and mechanisms.

To evaluate the prognostic utility of the model for OC patients, the OC patients were allocated into two risk groups. KM analysis revealed the low-risk group had the higher OS. ROC analysis demonstrated that the TRLs prognostic signature demonstrated potential in predicting OS. Subsequently, we confirmed the risk scores were the adverse prognostic factor. In addition, the nomogram enhanced the applicability of the prognostic model in clinical practice. The enrichment analysis was used to explore the mechanisms of prognostic TRLs. GO terms revealed these genes were observably related to fibrillar collagen trimer and collagen fibrill organization. The KEGG pathway contained proteoglycans in cancer and PI3K-Akt signaling pathway, etc. These biological processes and pathways likely contribute to the high-risk group towards poorer clinical OS.

We investigated the association between the characteristics of OC tumors and immune infiltration. The high-risk group had higher stromal score and estimate score, which might mean the activation of diverse biological behavior within the tumor microenvironment (46, 47). In addition, we find the levels of regulatory T cells (Tregs) were comparatively higher in the high-risk group, while the abundance of activated CD8 T cells and monocytes was the opposite by ssGESA. CIBERSORT analysis revealed that the high-risk group had significantly higher levels of M_2_ macrophages. Tregs are connected with dampening excessive immune activation and preserving immune homeostasis (48). Excessive activity of Tregs has the potential to promote the development of tumors (49). The strategic targeting of Tregs to restore a pro-inflammatory and immunogenic tumor microenvironment has gained increasing attention as an attractive approach for cancer treatment (50). CD8 T cells are the primary effector cells crucial for anti-tumor responses in immunotherapy. The phenomenon of CD8 T cell “exhaustion” frequently results in the loss of control and advancement of tumors (51). Monocytes, integral components of the mononuclear phagocyte system within the innate immune system, are crucial regulators of cancer initiation and progression. Distinct subsets of monocytes undertake diverse functions, contributing to both pro-tumoral and anti-tumoral immune responses (52). The expression of M_2_ macrophages is associated with a poor prognosis of OC (53). The study revealed that M_2_ macrophages stimulated the proliferation of OC cells, a process associated with the elevated expression of MMP9 (54). We observed discrepancies between the outcomes of ssGSEA and CIBERSORT analyses, which may point to contradictions. The varying levels of immune activity could potentially account for the poor prognosis observed in high-risk OC patients. It is noteworthy that the connection between the risk stratification of TRLs and immune response has not yet been verified. Elucidating the underlying mechanisms of this association is a valuable area for future investigation.

TMB is a critical measure of the tumor’s mutational load, which is translated into antigens that are presented to T cells. An elevated TMB can lead to the production of a greater number of neoantigens, increasing the chances of T cell recognition. This increased recognition may, in turn, enhance the efficacy of treatments involving immune checkpoint inhibitors by bolstering the immune system’s response against the tumor (55, 56). TMB levels are becoming widely acknowledged as a sensitive indicator predicting clinical responses to immunotherapy across diverse cancer types (57). Overall, TMB levels serve as a reflection of effective immune activation, with research indicating that higher TMB levels are associated with a greater likelihood of benefiting from immunotherapy (58). To investigate the essential roles of TMB in OC through somatic mutation analysis, we identified somatic mutations in 247 patients, with 137 (99.28%) in the high-risk group and 140 (99.29%) in the low-risk group. Consistent with findings from other studies, the TP53 gene had the highest mutation frequency (59). Moreover, The high-TMB group exhibited superior survival rates in OC, corroborating findings from the previous study (60). Interestingly, the high-TMB combined with the low-risk group demonstrated the highest OS rate, demonstrating that the low-risk group might derive more significant benefits from immunotherapy.

Additionally, we looked at patients’ responses to drug sensitivity, assessed through IC_50_ values. It is noteworthy that the above anti-tumor drugs were more sensitive to the low-risk group. In our subsequent analysis, we explored the correlation between PTPRD-AS1 expression and the sensitivity to the above anti-tumor drugs. Our findings indicated that patients with elevated PTPRD-AS1 levels exhibited a significantly better therapeutic response to Rapamycin and Dasatinib compared to those with lower levels. Rapamycin, a multifaceted immunosuppressant, has demonstrated therapeutic potential in the treatment of OC. Its antitumor activity is mediated through the inhibition of the mTOR signaling pathway, a critical cellular regulator of growth and proliferation (61, 62). Dasatinib, a potent tyrosine kinase inhibitor, has exhibited antitumor effects against OC (63). Collectively, these results indicate that patients with elevated levels of PTPRD-AS1 may respond more favorably to the therapeutic effects of Rapamycin and Dasatinib.

We verified six prognostic genes by qRT-PCR. The PTPRD-AS1, SPAG5-AS1, CHRM3-AS2, AC074286.1, FAM27E3 and AC018647.3 expression levels were significantly over-expressed in OC cell lines (SKOV3, A2780, CAOV3) in comparison to normal IOSE-80 cells. Notably, PTPRD-AS1 knockdown decreased cell proliferation, migration, and invasion in OC. In addition, we found a significant correlation between elevated PTPRD-AS1 expression and a poorer prognosis. And, PTPRD-AS1 was closely related to the immune cells. These results demonstrate that PTPRD-AS1 might serve as an efficient biomarker in OC.

Our study holds distinctive advantages. First, our prognostic model allows for a more nuanced understanding of the interactions between TRLs and patient outcomes. The risk of disease progression and prognosis for patients is evaluated by integrating clinical data with the expression levels of TRLs. Second, our model proposes a high-low risk stratification of patients and assesses their susceptibility to anti-cancer drugs as well as the efficacy of immunotherapy, which can facilitate more accurate treatment approaches and follow-up plans, providing the basis for personalized management and treatment strategies. Furthermore, our research has uncovered a novel biomarker for OC, PTPRD-AS1, which could be instrumental in identifying high-risk populations. It has the potential to enhance both the sensitivity and specificity of early diagnostic approaches, thereby improving the detection rate among at-risk individuals.

There are still some limitations to our study. First, aside from PTPRD-AS1, the remaining 5 TRLs need further experimental verification to confirm their roles. Second, our analysis was confined to the TCGA database, which means that our TRLs prognostic model needs to be validated using external datasets and ideally tested to test the predictive power of the model in a multicenter, large-scale clinical trial. In addition, achieving personalized precision studies depends on the integration of varied datasets, including clinical and multi-omics study data. Undoubtedly, our future research will be conducted in subsequent papers, including *in vitro* and *in vivo* experimental studies to better determine the capabilities of TRLs and mitigate the development of OC. We also need to collect blood and tissue samples from OC patients and integrate metabolomics and proteomics for comprehensive detection and analysis. This study is the first to verify the therapeutic potential of PTPRD-AS1 in OC. However, the precise mechanism by which PTPRD-AS1 influences prognosis, particularly its relationship with immune cell infiltration, remains to be elucidated. To explore this, We plan to examine the transcriptional effects of PTPRD-AS1 by conducting an RNA sequencing (RNA-seq) analysis. This will be complemented by the GO analysis to identify and characterize the functional roles of differentially expressed TRGs, providing insights into the mechanism of PTPRD-AS1. We will conduct flow cytometry assays to evaluate the impact of PTPRD-AS1 knockdown on the expression of OC cell surface markers. Additionally, we will co-culture these cells with peripheral blood mononuclear cells (PBMCs) or tumor-specific T cells to measure cytotoxic effects and the proliferation of immune cells. Immunohistochemical staining will be utilized to examine the spatial distribution and concentration of immune cells within tumor tissues, including CD8+ T cells, Monocytes, and Tregs. It is important to note that this study does not include drug-related validation. Consequently, future research will focus on pharmacokinetic studies to assess drug delivery, distribution, and accumulation of Rapamycin and Dasatinib in OC tissues.

## Data Availability

The original contributions presented in the study are included in the article/**Supplementary Material**. Further inquiries can be directed to the corresponding authors.
