# Supplementary material for: Identification of telomere-related lncRNAs and immunological analysis in ovarian cancer
Source: Front Immunol. 2024 Sep 17;15:1452946. doi: 10.3389/fimmu.2024.1452946 (PMC11442270; doi:10.3389/fimmu.2024.1452946)
Supplement: Supplementary file 1 [file DataSheet1.docx]

Supplementary Material

## Supplementary Figure 1


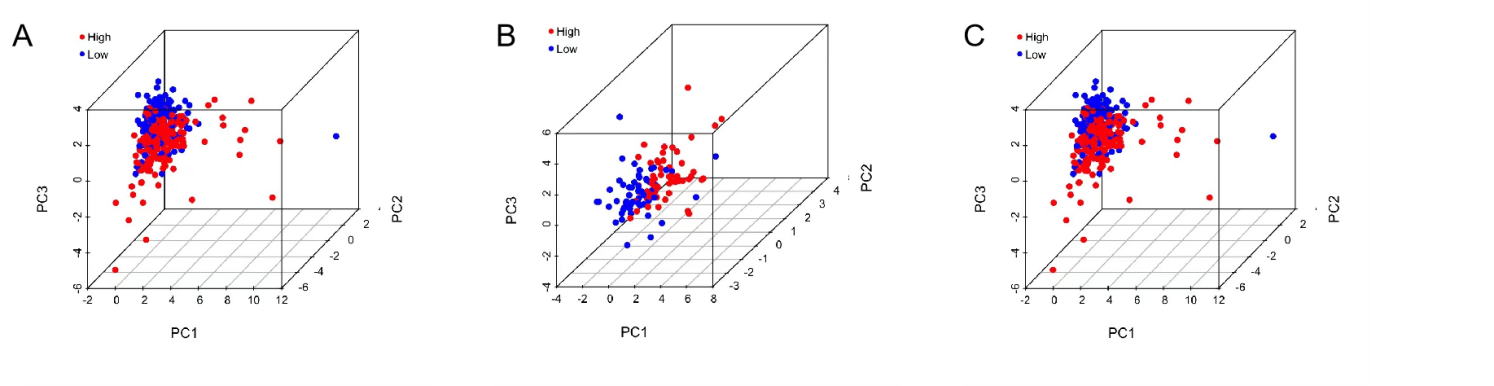


## Supplementary Figure 1. Principal components analysis between low‐ and high‐risk groups with different data sets. (A) The training cohort. (B) The testing cohort. (C) The entire cohort.

## Supplementary Figure 2


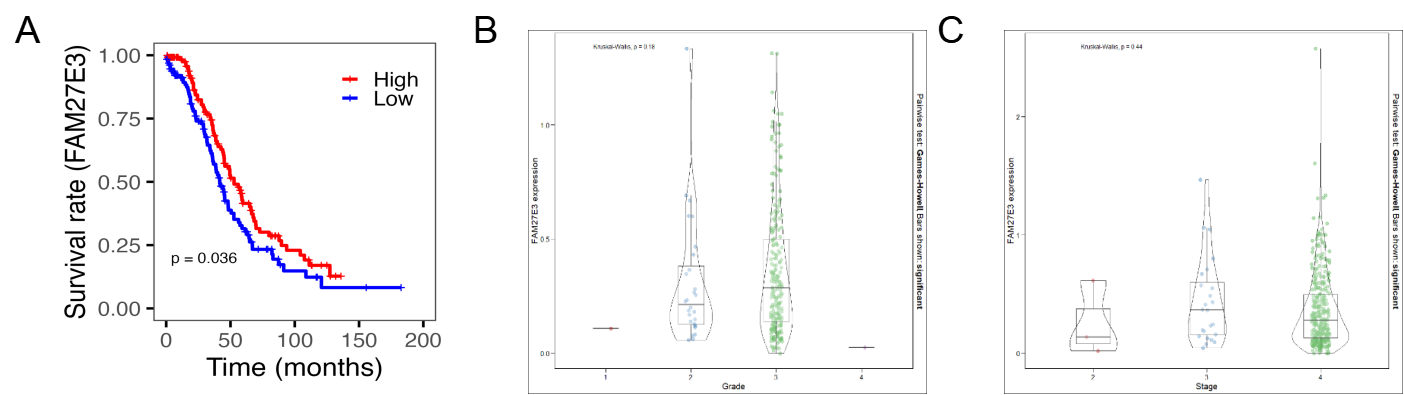


**Supplementary Figure 2.** FAM27E3 associated with OC prognosis. (A) Differences in prognosis between high and low FAM27E3 patients. (B) The levels of FAM27E3 in among different stages in OC. (C) The levels of FAM27E3 in among different grades in OC.

## Supplementary Figure 3


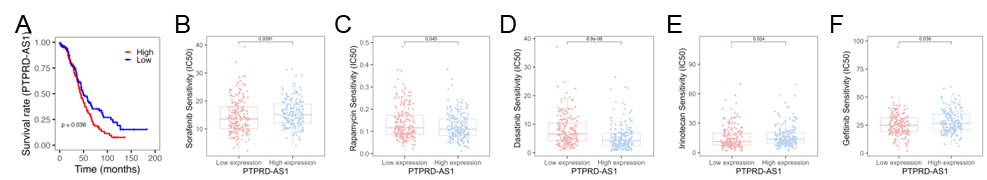


**Supplementary Figure 3.** PTPRD-AS1 associated with OC prognosis and chemotherapy response. (A) Differences in prognosis between high and low PTPRD-AS1 patients. (B-F) The estimated IC_50_ values of drugs in high and low PTPRD-AS1 groups.

**Supplementary Figure 4**


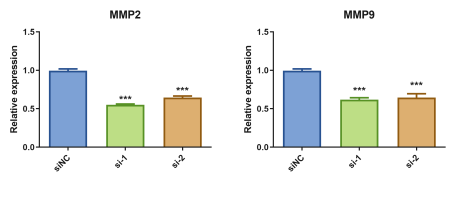


**Supplementary Figure 4.** The levels of MMP2 and MMP9 in transduced CAOV3 cells.
