# Supplementary material for: Identification of telomere-related lncRNAs and immunological analysis in ovarian cancer
Source: Front Immunol. 2024 Sep 17;15:1452946. doi: 10.3389/fimmu.2024.1452946 (PMC11442270; doi:10.3389/fimmu.2024.1452946)
Supplement: Supplementary file 2 [file Table1.docx]

Supplementary Table 1. Clinical features of all individuals encompassed in this work.

| **Variable** | **Entire cohort (n = 370)** | **Training cohort (n = 259)** | **Testing cohort (n = 111)** |
| --- | --- | --- | --- |
|  | **Number (%)** | **Number (%)** | **Number (%)** |
| **Age** |  |  |  |
| ≤65 | 114 (69.19%) | 82 (31.66%) | 32 (28.83%) |
| >65 | 256 (30.81%) | 177 (68.34%) | 79 (71.17%) |
| **stage** |  |  |  |
| I | 0 (0%) | 0 (0%) | 0 (0%) |
| II | 22 (5.95%) | 19 (7.34%) | 3 (2.70%) |
| III | **290 (78.38%)** | **202 (77.99%)** | **88 (79.28%)** |
| IV | **56 (15.14%)** | **36 (13.90%)** | **20 (18.02%)** |
| Unknown | **2 (0.54%)** | **2 (0.77%)** | 0 (0%) |
| **Grade** |  |  |  |
| I | **1(0.27%)** | **1(0.39%)** | **0** |
| II | **42(11.35%)** | **28(10.81%)** | **14(12.62%)** |
| III | **317(85.68%)** | **223(86.1%)** | **94(84.68%)** |
| IV | **1(0.27%)** | **1(0.39%)** | **0** |
